# A Decade of Progress Accelerating Malaria Control in Mali: Evidence from the West Africa International Center of Excellence for Malaria Research

**DOI:** 10.4269/ajtmh.21-1309

**Published:** 2022-10-13

**Authors:** Seydou Doumbia, Nafomon Sogoba, Mahamadou Diakite, Mahamoudou Toure, Moussa Keita, Drissa Konaté, Sory I. Diawara, Ayouba Diarra, Daouda Sanogo, Fousseyni Kane, Seidina A. S. Diakite, Karim Traore, Sidibé M’Baye Thiam, Sékou F. Traoré, Idrissa Cisse, Jules Mihigo, Mamadou B. Coulibaly, Djeneba Dabitao, Michael Alifrangis, Alyssa E. Barry, Günter C. Müller, John C. Beier, Jeffrey G. Shaffer

**Affiliations:** ^1^ Malaria Research and Training Center, University of Sciences, Techniques and Technologies of Bamako, Bamako, Mali;; ^2^ University Clinical Research Center, University of Sciences, Techniques and Technologies of Bamako, Bamako, Mali;; ^3^ National Malaria Control Program, Ministry of Health, Bamako, Mali;; ^4^ U.S. President’s Malaria Initiative, United States Agency for International Development Office, Bamako, Mali;; ^5^ Centre for Medical Parasitology, Department of Immunology and Microbiology, University of Copenhagen, Copenhagen, Denmark; Department of Infectious Diseases, Copenhagen University Hospital (Rigshospitalet), Copenhagen, Denmark;; ^6^ School of Medicine, Deakin University, Geelong, Australia;; ^7^ Miller School of Medicine, University of Miami, Miami, Florida;; ^8^ School of Public Health and Tropical Medicine, Tulane University, New Orleans, Louisiana

## Abstract

This article highlights over a decade of signature achievements by the West Africa International Centers for Excellence in Malaria Research (WA-ICEMR) and its partners toward guiding malaria prevention and control strategies. Since 2010, the WA-ICEMR has performed longitudinal studies to monitor and assess malaria control interventions with respect to space-time patterns, vector transmission indicators, and drug resistance markers. These activities were facilitated and supported by the Mali National Malaria Control Program. Research activities included large-scale active and passive surveillance and expanded coverage of universal long-lasting insecticide-treated bed nets and seasonal malaria chemoprevention (SMC). The findings revealed substantial declines in malaria occurrence after the scale-up of control interventions in WA-ICEMR study sites. WA-ICEMR studies showed that SMC using sulfadoxine‐pyrimethamine plus amodiaquine was highly effective in preventing malaria among children under 5 years of age. An alternative SMC regimen (dihydroartemisinin plus piperaquine) was shown to be potentially more effective and provided advantages for acceptability and compliance over the standard SMC regimen. Other findings discussed in this article include higher observed multiplicity of infection rates for malaria in historically high-endemic areas, continued antimalarial drug sensitivity to *Plasmodium falciparum*, high outdoor malaria transmission rates, and increased insecticide resistance over the past decade. The progress achieved by the WA-ICEMR and its partners highlights the critical need for maintaining current malaria control interventions while developing novel strategies to disrupt malaria transmission. Enhanced evaluation of these strategies through research partnerships is particularly needed in the wake of reported artemisinin resistance in Southeast Asia and East Africa.

## OVERVIEW OF THE WEST AFRICA INTERNATIONAL CENTERS OF EXCELLENCE FOR MALARIA RESEARCH

Recent and wide deployment of malaria control interventions has contributed to substantial reductions in malaria morbidity and mortality in sub-Saharan Africa.^1^^,^^2^ Yet, the region continues to bear the brunt of the global malaria burden, accounting for over 95% of the malaria cases and deaths worldwide.^3^ Scale-up initiatives for a host of malaria control strategies have been highly successful, with considerable progress in long-lasting insecticide-treated bed nets (LLINs), intermittent presumptive treatment of pregnant women (IPTp), seasonal malaria chemoprevention (SMC), and artemisinin-based combination therapy (ACT). Despite this progress, high levels of sustained malaria continue to occur in Sub-Saharan Africa.^4^^–^^7^ In 2010, the National Institute of Allergy and Infectious Diseases (NIAID) established a network of 10 International Centers of Excellence for Malaria Research (ICEMRs) to support multidisciplinary research in diverse epidemiologic settings representing malaria-endemic regions worldwide.^8^ ICEMR studies focused on the complex interactions among the human host, parasite, and mosquito vector populations that may be differentially affected by control interventions in different epidemiological settings. The West Africa ICEMR (WA-ICEMR) was established to advance ICEMR initiatives in Mali by evaluating the impacts of differential malaria endemicity and investigating the effectiveness of the implementation practices related to those policies.^9^^,^^10^ The WA-ICEMR investigators sought to understand the variable effectiveness of deployed malaria control interventions in Mali and vector populations in different ecological settings and provide evidence-based approaches for guiding public policy in Mali and across West Africa. This article highlights signature WA-ICEMR achievements and findings over the past decade toward achieving these objectives.

### Ethics statement.

Several studies described here used data from published works, and detailed ethics statements are available within those articles. Study protocols were approved by the Ethical Committee of the Faculty of Medicine, Pharmacy, and Odontostomatology for the University of Sciences, Techniques, and Technologies of Bamako. Treatment and patient care were performed by trained personnel at Mali public health units or the Malian National Malaria Control Program (NMCP). Clinical trials conducted at the Dangassa and Koulikoro study sites were registered at ClinicalTrials.gov (registration numbers NCT04149106 and NCT02086877, respectively).

## RESEARCH IN THREE COMMUNITIES REPRESENTING DIFFERENT MALARIA ECOSYSTEMS IN MALI

Malaria is highly endemic to Mali, particularly in its central and southern regions, where over 90% of its population resides. Malaria prevalence among children younger than 5 years old declined from 47% in 2012 to 19% in 2018.^11^ In collaboration with the U.S. President’s Malaria Initiative (PMI) and other donors, benchmarks have been set for reducing Mali’s malaria burden by 50% by 2030. More ambitious malaria elimination goals have been set forth by the NMCP and Mali’s Ministry of Health to eradicate malaria in Mali by 2030.^12^ Among the landmark achievements of the WA-ICEMR were its large-scale surveillance and implementation studies. These studies were carried out at three primary field sites with differential malaria endemicity, implementation approaches, and control strategies. The first site, Dangassa, is a rural village situated along the Niger River. The second site, Dioro, lies in the inland delta region of the Niger River and was one of eight Mali intervention areas for the United Nations Millennium Villages Project (MVP).^13^^–^^15^ Launched in 2005, the MVP was an ambitious, 10-year initiative to address agriculture, environmental restoration, primary education, primary healthcare, and local infrastructure in rural areas in sub-Saharan Africa with high rates of malnutrition.^16^^,^^17^ MVP’s malaria control interventions, including universal coverage of LLINs, active case detection (ACD), IPTp, and universal health coverage with free treatment at local health facilities were implemented in Dioro between 2007 and 2014.^18^ The third site was in Koulikoro District, a North Savanna area, where indoor residual spraying (IRS) campaigns were implemented for over 8 years. The general characteristics and ecological settings for these sites are provided in Table 1.

**Table 1:** WA-ICEMR study site characteristics

Characteristic	Mali study site		
	Dangassa	Koila, Dioro	Sirakorola, Koulikoro
Date of WA-ICEMR cohort enrollment	2012	2012	2017
Proximity to Bamako	80 km Southwest	313 km Northeast	112 km Northeast
Climate/ecology	North savanna 2–4 km from River Niger Extensive seasonal rainfall (June to October)	Sahelian plus River flood plain, irrigation (July to October)	Dry semiarid/Sahelian (June to October)
Population (inhabitants)	7,800	6,000	7,000
Urbanicity	Rural	Rural	Semiurban
Land use	Agriculture, gold mining, fishing	Irrigated rice cultivation, cattle farming, fishing	Agriculture, cattle farming
Length of Malaria transmission	June–December	July–October	June–October
Peak of malaria transmission	October	October	October
Malaria Control Interventions			
LLINs as universal coverage	Introduced in 2015	MVP, 2007–2014, then 2016	Introduced in 2015
SMC	Introduced in 2015	Introduced in 2016	Introduced in 2016
Antimalaria treatment (ACT) policy	Free treatment of all RDT positive patients from ICEMR cohorts	Free treatment of all RDT positive patients from ICEMR cohorts	Free treatment of all RDT positive patients from ICEMR cohorts
IRS	No	No	2008–2016

IRS = indoor residual spraying; LLIN = long-lasting insecticidal nets; MVP = Millennium Villages Project; RDT = rapid diagnostic test; SMC = seasonal malaria chemoprevention; WA-ICEMR = West Africa International Centers for Excellence in Malaria Research. Calendar months January, June, July, August, October, and December abbreviated as Jan., Jun., Jul., Aug., Oct., and Dec., respectively. Malaria defined as uncomplicated *P. falciparum* symptomatic infection.

Further details for these study sites were described previously in recent works.^18^^–^^22^

## INSIGHTS FROM RESEARCH ACTIVITIES HAVE ENRICHED THE UNDERSTANDING OF IMPLEMENTED MALARIA CONTROL INTERVENTIONS

### Trend analyses in malaria epidemiological factors from WA-ICEMR cohorts following the implementation of current control strategies.

The WA-ICEMR studies performed large-scale ACD and passive case detection (PCD, respectively) surveillance activities, including rolling cohort studies that included over 1,400 subjects at each site.^19^ The ACD was used to determine the prevalence of asymptomatic *Plasmodium (P.) falciparum* infection through cross-sectional surveys on a biannual basis corresponding to the beginning and end of peak transmission periods. Year-round PCD was performed at community health centers for each field site. The primary outcome for PCD was uncomplicated *P. falciparum* malaria, which was tested using rapid diagnostic tests (RDTs) and microscope slides (blood smears). Local health staff provided treatment of presenting symptomatic subjects free of charge as per Mali national policy or as ancillary care for participation. Samples were collected using blood smears for microscopy diagnostics, and filter papers were used for parasite genotyping and evaluating *P. falciparum* drug-resistance molecular markers.^23^^,^^24^

### Temporal changes in the prevalence of asymptomatic *P. falciparum* infection.

**Figure 1.**
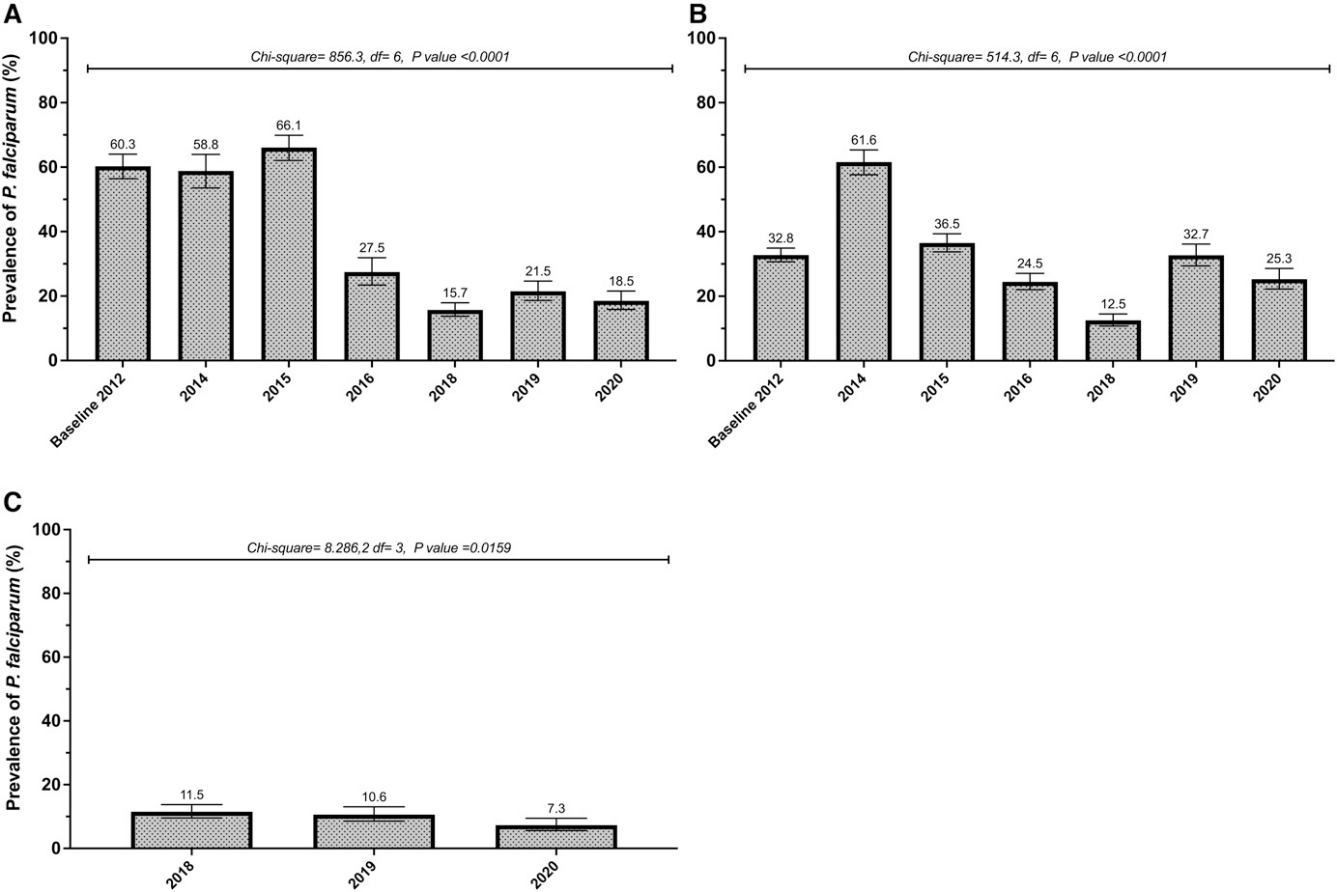
Temporal changes in overall prevalence of *P. falciparum* infection (based on microscopy) at the end of the transmission season (October). (**A**) Dangassa and (**B**) Dioro, from 2012 to 2020, and (**C**) Koulikoro, from 2018 to 2020. Surveys were not performed in 2013 and 2017 at the end of the transmission season in Dangassa and Dioro, and surveys started in 2018 at the Koulikoro site. The error bars indicate the standard deviation for each estimate.

Declines in the overall prevalence of asymptomatic *P. falciparum* infection were observed for the Dangassa and Dioro study sites following the scale-up of malaria control interventions (Figure 1).

In Dangassa, the prevalence of asymptomatic infection decreased from 60.3% in 2012 to just 18.5% in 2020.^25^ In Dioro, prevalence rates abruptly increased from 32.8% in 2012 to 61.6% in 2014 (following the discontinuation of universal healthcare access supported by the MVP). In Koulikoro, the newest WA-ICEMR site established in 2018, the prevalence of asymptomatic infection declined from 11.5% in 2018 to 7.3% in 2020. The substantial declines in the overall prevalence of asymptomatic *P. falciparum* infection rates at the Dangassa and Dioro sites are likely attributable to the implementation of SMC (starting in 2015) and universal coverage of LLINs (also starting in 2015). The expansion of SMC to older children in 2019 (through the WA-ICEMR implementation research carried out in collaboration with the NMCP) may have contribute to the decline of the prevalence in Koulikoro study site.

### Age-specific declines in the prevalence of *P. falciparum* infection.

**Figure 2.**
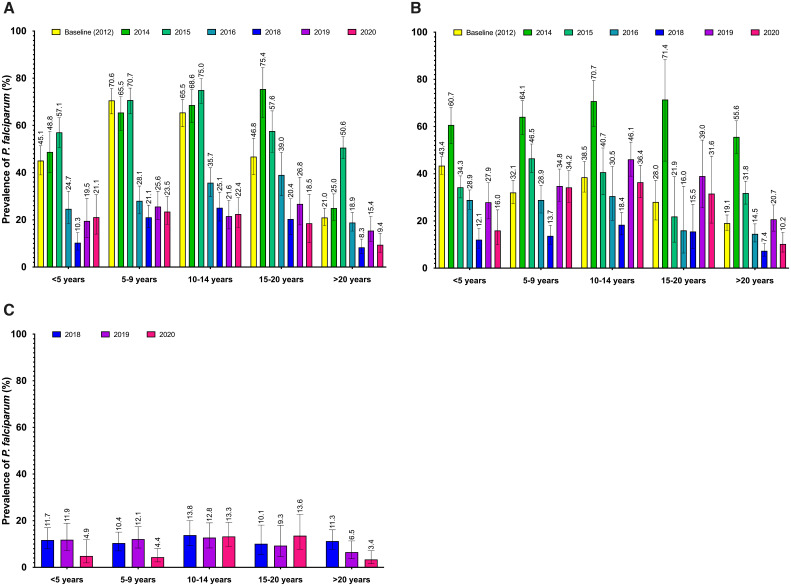
Temporal changes in age-specific prevalence of *P. falciparum* infection (based on microscopy) at the end of the transmission season (October). (**A**) Dangassa and (**B**) Dioro, from 2012 to 2020, and (**C**) Koulikoro, from 2018 to 2020. The error bars indicate the standard deviation for each estimate.

Figure 2 displays the temporal trends in the age-specific prevalence rates of asymptomatic *P. falciparum* infection for the three study sites.

Overall, significant declines in the prevalence of asymptomatic *P. falciparum* infection were observed among all age groups after 2015 (when universal LLINs coverage and SMC implementation started in Dangassa and in Dioro). At the Dangassa site, prior to 2016, the highest prevalence was observed in older children (≥5 years old) reaching 75%. The prevalence of infection in children under 5 years old reached historically low levels in 2018 (10.3%, Figure 2A). More importantly, older children (≥5 years old) and subjects over 20 years old continued to carry malaria parasites, though the prevalences were generally lower after 20 years of age than those for younger age groups (peaking at 50.6% in 2015 and reaching a low of 8.3% in 2018 among subjects over 20 years old). Similar trends were observed at the Dioro and Koulikoro study sites (Figure 2B). Notably, an upsurge in 2014 (after withdrawal of the MVP malaria control intervention in the area) was followed by a sharp decrease after 2015 (SMC plus LLINs implementation) was observed in all age groups in Dioro. In the low transmission areas of Sirakorola, Koulikoro, a sharp decrease in the prevalence among children under 10 years old was observed in 2020 after SMC implementation study expanded the intervention to older children in the area (Figure 2C).

### Malaria incidence displays high seasonality.

At each study site, most symptomatic malaria cases detected through PCD were concentrated in five or six month periods. At the Dangassa and Dioro sites, malaria cases were highly seasonal and primarily occurred in more extended periods between June and December. At the Sirakorola, Koulikoro site, malaria cases were detected exclusively from July to November. Though incidence rates remained low, malaria continued to occur during the dry season at the Dangassa and Dioro sites, which was likely influenced by the dry season rice cropping cycle in Dioro and mosquito vector breeding habitats along the Niger River in Dangassa.

### Spatiotemporal heterogeneity of malaria distribution in relation with environmental and climatic conditions.

To better characterize the distribution of malaria and potential hot spots, spatial analyses were carried out to detect clusters of malaria cases over geographic space or time period. The geographic locations of human and vector habitats were mapped using high-resolution satellite imagery and probabilistic spatial models. One study revealed high levels of space-time clustering of *P. falciparum* infection in the dry and rainy seasons for both the Dangassa and Dioro sites.^19^ The most marked temporal clustering occurred for the Dioro site, where the prevalence of *P. falciparum* infection rose to 60% and remained high into the dry season of 2015. This finding is likely attributable to the stoppage of financial support in 2014 (May–June) for LLINs, ACT, and IPTp.^19^ In two other studies, relationships between environmental and climatic conditions and malaria incidence were analyzed. Functional generalized additive models (GAM) showed higher predicted malaria incidence rates 10–12 weeks following air humidity levels reaching over 65% and two or more consecutive rain episodes and mean wind speed under 1.8 m/second.^26^ Temperatures were directly associated with malaria incidence for each study site.^20^ Wind speed and river heights contributed to increasing malaria incidence in Dangassa, in contrast to humidity and vegetation at the Dioro site. Applied statistical models also showed that some observed meteorological conditions might assist in malaria outbreak detection.

### Monitoring malaria drug-resistance molecular markers and *P. falciparum* genetic diversity.

Drug resistance is among the most significant challenges and threats to malaria control. The diversity and the prevalence of *P. falciparum* drug-resistance molecular markers were assessed using next-generation sequencing (NGS) strategies to track drug-resistant malaria parasites in WA-ICEMR study sites.^23^ Additionally, *P. falciparum* isolates from malaria-infected children were tested for ex-vivo sensitivity of *P. falciparum* to standard antimalarial drugs used in Mali. The analysis of antimalarial drug-resistance molecular markers revealed a low prevalence of amodiaquine (AQ) resistance-associated mutation (Pfmdr1_86Y mutant parasite, 0.01%) and a high prevalence of Pfmdr1_Y184F, the lumefantrine-reduced susceptibility mutation (39.9%).^23^ Only two isolates from Dangassa exhibited the piperaquine (PQ)-resistance Exo-E415G mutation. No artemisinine resistance parasite genetic background (PGB) was detected in the genotyped isolates. Although a high level of codon substitutions in both the Pfdhfr and Pfdhp genes was found only one isolate presented the Pfdhfr_51R-59N-108I/Pfdhps_437G-540E quintuple substitution in Dangassa. High *P. falciparum* diversity was apparent but did not correspond with well-defined genetic aggregation.^23^ Ex-vivo studies showed high sensitivities of *P. falciparum* isolates to lumefantrine, dihydroartemisinin (DHA), and PQ (100% each), with lower observed sensitivities for quinine (74.3%), chloroquine (87.8%), AQ (97.2%), and mefloquine (98.7%).^24^ These results suggest that current antimalaria drugs used for control intervention in Mali are relevant and highlight the need for routine drug-resistance monitoring.

## IMPLEMENTATION RESEARCH FOR ENHANCING CURRENT SMC INTERVENTIONS IN MALI

### Assessing the effectiveness of DHA-PQ as a potential alternative to the SP-AQ for SMC drug regimen.

**Figure 3.**
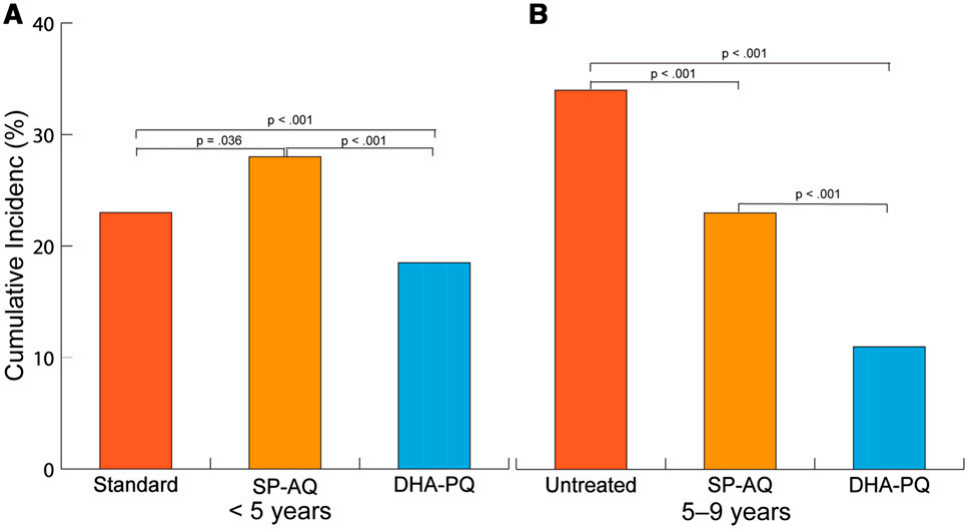
Standard = comparison arm 1 villages, where SP-AQ provided to children aged between 3 months and 5 years only (Standard SMC in Mali). SP-AQ sulfadoxine-pyrimethamine plus amodiaquine, comparison arm 2 villages where SP-AQ provided to children aged between 3 months and 9 years (SMC extended to older children with SP-AQ). DHA-PQ = dihydroartemisinin plus amodiaquine, comparison arm 3 villages, where DHA-PQ provided to children aged between 3 months and 9 years old (SMC extended to older children with DHA-PQ). Panel A shows *Plasmodium falciparum* malaria incidence rates among children under 5 years old by study arm. Panel B shows *Plasmodium falciparum* malaria incidence rates by study arm among children 5–9 years old. This figure appears in color at www.ajtmh.org.

Current national policy in Mali provides SMC coverage for children between 3 months and 5 years old. WA-ICEMR investigators sought to determine whether expanding SMC coverage to between 5 and 9 years old using two treatments: sulfadoxine-pyrimethamine (SP) plus AQ, or SP-AQ (the standard drug regimen for SMC), and DHA plus PQ, or DHA-PQ (as an alternative to SP-AQ). These studies were performed at the Koulikoro field site and included 6,217 children (3,380 children aged under 5 years and 2,837 children between 5 and 9 years old) from 49 villages and nine health catchments. The catchments were randomly allocated according to three treatment strategies: SP-AQ in children between 3 months and 5 years old (standard of care); SP-AQ in children between 3 months and 9 years old; and DHA-PQ in children between 3 months and 9 years old. SP-AQ and DHA-PQ were both highly effective in preventing malaria in children between 5 and 9 years old (Figure 3).

Notably, DHA-PQ was preferred over the standard SP-AQ regimen in terms of taste. Malaria incidence was between 2.0 and 3.3 times higher in the control group (children between 3 months and 5 years old) than in children between 3 months and 10 years of age receiving SP-AQ or DHA-PQ. Malaria parasitemia also showed a significant risk reduction for asymptomatic malaria, decreasing in children under 15 years old by 65% and 81% for those receiving SP-AQ and DHA-PQ respectively.

### Impact of expanding SMC to older children with an additional round of SMC provision.

In another WA-ICEMR SMC study, a cohort of 350 children between 5 and 14 years of age was randomly assigned to five monthly rounds of SP-AQ (the standard SMC regimen for children between 3 months and 5 years old) and compared with a control group of untreated children.^27^ The study design included five monthly treatment cycles instead of the current standard of four monthly treatment rounds. Children between 5 and 14 years old who received SMC were 78% less likely to have malaria parasitemia and 85% less likely to have anemia than children in the same age group not receiving SMC (adjusted odds ratio [aOR], 95% CI: 0.22 [0.11–0.42]) and 0.14 [0.07–0.28], respectively). Prevalence rates in December (the fifth and final treatment cycle) were significantly lower in treated children, suggesting that the additional round of SMC was highly efficacious. Limited data (not shown) comparing malaria occurrence among subjects that received all five treatment cycles versus subjects that received four cycles further supported this claim. Overall, ≈34.7% of new *P. falciparum* asymptomatic infections were averted by providing SMC to children 5–9 years old.^27^

## WA-ICEMR ENTOMOLOGICAL STUDIES AND THE POTENTIAL VALUE OF VECTOR CONTROL AND ROUTINE MALARIA SURVEILLANCE

### Assessing changes in malaria vector population and transmission patterns relative to ongoing malaria control interventions.

Like the aforementioned malaria prevalence and incidence studies, malaria entomological parameters were measured through biannual surveys at the beginning and end of the transmission season to determine changes in transmission intensity that were potentially attributable to control interventions. During each survey, mosquito collection was performed using pyrethrum spray-catch and human landing catch approaches at both indoor and outdoor locations. *Anopheles* (*A*.) *gambiae* complex species were identified by polymerase chain reaction (PCR) tests, and enzyme-linked immunosorbent assays (ELISAs) were used to determine the origin of mosquito blood meals and the presence of *P. falciparum* sporozoite infections.^22^ Entomological infection rates (EIRs) were calculated as human biting rates times the total number of positive ELISA results divided by the total number of samples tested. Malaria vector populations differed slightly by study site.^22^ In Dangassa, *A. coluzzii* was the predominant species (91.4%), followed by *A. gambiae* (8.0%) and *A. arabiensis* (<1%). In Dioro, *A. coluzzii* represented 99% of the vectors, followed by *A. arabiensis* (0.8%) and *A. gambiae* (0.2%). In Koulikoro, vector composition consisted of *A. coluzzii* (96.0%), *A. gambiae* (2.0%) and *A. arabiensis* (2.0%). Human blood indices (HBI) were significantly higher for Dangassa and Koulikoro (79.4% and 74.3%, respectively) than Dioro (15.9%). The average monthly human biting rate (MHBR) was 2.3 times higher in Dioro (69.8 bites/person/month) than in Dangassa (30 bites/person/month).^22^ The MHBR was lower in Koulikoro compared with the other two sites, even following the discontinuation of IRS (8.1 bites/person/month). However, there was no significant difference in overall sporozoite infection rates (SIR) between the study sites (2.9% [95% Bayesian credible interval [BCI]: 2.3–3.6] versus 2.6%, [95% BCI: 2.2–3.1] for Dangassa and Dioro, respectively). Decreasing temporal trends were observed in Dangassa and Dioro in both vector densities and EIRs, which was consistent with observed declines in asymptomatic infection prevalence rates. The EIRs peaked in August 2014 and generally declined through the most recent reporting period in October 2019. The WA-ICEMR data sources offered the opportunity to assess patterns in vector species composition, feeding behavior, and their influence on indoor and outdoor vector transmission settings. The Dangassa and Dioro sites were comparable with respect to both indoor and outdoor mosquito feeding rates.^22^ The WA-ICEMR data sources also offered the opportunity to assess patterns in vector species composition, feeding behavior, and their influence on indoor and outdoor vector transmission settings. The Dangassa and Dioro sites were comparable with respect to both indoor and outdoor mosquito feeding rates.^22^

### Dry season vector ecology studies for enhancing outdoor-based vector control interventions.

The WA-ICEMR focused on the ecology of outdoor *Anopheles* malaria vector populations as vector control has traditionally concentrated on indoor transmission, which may be insufficient for controlling malaria. Key ecological gaps occurring outside villages that may contribute to understanding transmission dynamics include sources and survival strategies of vector populations during extended dry seasons. Malaria vector ecology studies used ground searches, camera-mounted drones, and satellite imagery to identify and define the extent of the dry season and vector habitats. The WA-ICEMR studies characterized dry season refuges for *A. coluzzi* and *A. gambiae* and demonstrated their dispersal at the onset of the rainy season. Favorable, natural outdoor resting sites were identified within the riverine, particularly at its edges and in its dense undergrowth. *A. gambiae sensu lato* (s.l.) were found to shift or adjust their resting sites during the day according to sun movement and exposure. Sugar sources were identified to determine factors associated with malaria vector survival during the dry season, showing that flowering plants favored female populations.^28^ Invasive and ornamental or agricultural plants were identified and found to be highly attractive to mosquitoes, where they provided sugar sources and sites for diurnal mosquitos resting in the dry season. It is worth mentioning that leaf phenologies of invasive species were successful because, unlike native species, they did not lose their leaves during the dry season. Additional sugar meal sources for *A. gambiae* included honeydew melons throughout the dry season. The studies also found, to our knowledge for the first time, that both *A. coluzzi* and *A. gambiae* feed extensively on birds in isolated, uninhabited natural habitats. The study showed that large numbers of marked mosquitoes were able to cross the Niger River (over 1.0 km wide) from the refuges and ended up in a riverine forest (1.2 km wide), lagoon (1.2 km wide), fishing hamlet (1.5 km wide), and a nearby Kenieroba village (2.0 km wide). These factors likely represent sources for dry season malaria transmission.

#### Prevalence of insecticide resistance in major malaria vector species.

Knowledge regarding insecticide resistance is critical for successful malaria vector control. The WA-ICEMR assessed *A. gambiae* s.l. population resistance to pyrethroids in different ecological settings. The World Health Organization’s (WHO) standard bioassay test was used to assess *A. gambiae* s.l. susceptibility to pyrethroids. Biochemical synergist assays were conducted with piperonyl butoxide (PBO), S,S,S-tributyl phosphorotrithioate, and diethyl maleate. L1014F, L1014S, and N1575Y knockdown resistance (kdr) mutations were investigated using TaqMan genotyping.^29^ The WHO’s standard bioassay tests showed high phenotypic resistance to pyrethroids (reaching 13–50% mortality rates) in Koulikoro, where IRS was performed).^29^ Molecular markers for kdr were present in the three malaria vector species (*A. gambiae, A. coluzzii*, and *A. arabiensis*) at all three study sites.^30^ Preexposure to synergists showed partial restoration of susceptibility with PBO, the inhibitor of cytochrome P450 enzymes, which indicated the presence of metabolic resistance. Phenotypic resistance was also observed with carbamates to the three species, which was consistent with acetylcholinesterase (ace-1^R^) target-site mutation in all vector species studied.^31^ The three species of *A. gambiae* s.l. were fully susceptible to the organophosphates (pirimiphos-methyl) at each study site.^29^ Target-site mutation and metabolic resistance mechanisms each played roles in resistance patterns observed in the three species of *A. gambiae* s.l. and the kdr-west (kdr-w) mutation. The WA-ICEMR observed and reported for the first time in Mali the presence of the N1575Y and the kdr-east (kdr-e) mutation in *A. arabiensis*.^30^ The PBO provided a better partial restoration of susceptibility to pyrethroids, suggesting that it may improve the efficacy of LLINs.^30^^,^^32^ Susceptibility to pirimiphos-methyl was confirmed and could be used as part of a rotation strategy for resistance management.

### Performance of integrated vector control tools (LLINs and IRS).

Malaria vector control relies heavily on LLINs and IRS in selected districts. As part of strengthening vector control strategies in Mali’s Koulikoro District, the NMCP strategically implemented IRS with LLINs with high coverage. Due to the increased reports of vector resistance to both pyrethroid and carbamates, IRS shifted to the use of pirimiphos-methyl, (an organophosphate), in Koulikoro, between 2015 and 2016. The effect of IRS on malaria transmission was assessed by comparing key entomological parameters between Koulikoro, (where IRS was implemented), and its neighboring district (where IRS was not previously used).^29^ WA-ICEMR studies showed a rise in malaria prevalence of four times higher for the non-IRS areas than the IRS areas.^21^ Also, overall malaria incidence rates were two times higher in non-IRS areas than IRS areas. Malaria vector species in both non-IRS and IRS areas were composed primarily of *A. coluzzii*, *A. gambiae*, and *A. arabiensis* and were highly resistant to pyrethroids and carbamates and susceptible to pirimiphos-methyl.^29^ IRS using pirimiphos-methyl significantly reduced entomological parameters of malaria transmission.

## SIGNIFICANCE OF RESEARCH FINDINGS FOR IMPROVING MALARIA CONTROL OUTCOMES

Mali is highly endemic to malaria and benefits from strong commitments with significant investments by NMCP and its partners including U.S. PMI, Global Funds, and other donors to scale-up proven malaria control interventions. The WA-ICEMR studies offer unique opportunities for evaluating and providing evidence-based approaches for improving malaria control interventions in a diverse set of environmental and ecological settings. The diverse settings here permitted comparisons according to environmental factors that are often possible only through multicountry studies. For instance, Dangassa straddles the Niger River and maintains variable of mosquito breeding levels within the site. Site-to-site variability was also particularly helpful in applying meaningful comparative approaches and analyses. Entomological surveillance was also a core part of the WA-ICEMR activities, particularly for monitoring insecticide resistance, which is critical for early resistance detection. This groundwork provides opportunities for leveraging these efforts to develop more sophisticated data systems such as early warning and prediction systems. Another particular advantage of the study sites and timeline for the studies here is their pre- and post-SMC coverage periods. Because national policy covers SMC for children under 5 years of age, the WA-ICEMR was able to leverage large-scale governmental resources and initiatives.

### Trend analyses display substantial declines in malaria infection and transmission patterns related to control interventions.

Intensification of malaria control interventions including universal LLINs coverage and SMC implementation may have contributed to the significant declines in asymptomatic *P. falciparum* prevalence rates in all age groups. However, the persisting higher asymptomatic *P. falciparum* malaria prevalence rates among children between 5 and 9 years old in the high-endemic settings of Dangassa and Dioro may indicate long-term trends and could pose a significant threat to disrupting malaria should this burden continue. Extending SMC coverage to children at least 5 years old and increasing the number of monthly treatment cycles (from four to five) was highly influential in our studies. These strategy offer great opportunities to better control malaria burden persistence in children transitioning out of the standard SMC coverage, particularly in stable, long-transmission areas. School-based delivery strategies can be considered for improving SMC coverage in older children. The findings here also accentuate the need to maintain the intensification of malaria control interventions to reach the NMCP goal of elimination. The WA-ICEMR studies performed in Koila, Dioro showed how the abrupt stoppage of malaria control intervention could be highly consequential. Malaria transmission and morbidity rose by more than 80% in October 2014 when the MVP project (which provided universal coverage for LLINs, ACD, IPTp, and universal health coverage with free treatment at local health facilities) ended.^19^ These dramatic increases were mitigated with the return of the national malaria control activities in 2016, including universal LLINs and SMC through the coordinated efforts between the NMCP and WA-ICEMR.

### DHA-PQ appears to be a viable alternative to SP-AQ as an SMC drug treatment strategy.

DHA-PQ is a promising ACT recently endorsed by the WHO as a potential alternative treatment of uncomplicated *P. falciparum* malaria in Africa. Several recent clinical trials revealed that DHA-PQ is safe and efficacious for treating uncomplicated malaria.^33^ Recent WA-ICEMR studies also suggest that DHA-PQ is more effective than SP-AQ in preventing malaria, and showed additional benefits, including a more acceptable taste, increased treatment compliance, and fewer side effects. However, it is worth mentioning that the *kelch13* gene has been detected in sub-Saharan Africa,^34^ which raises concern that widespread use of DHA-PQ may increase the risk of artemisinin resistance. Regular monitoring of molecular resistance markers for alternative SMC regimens is vital to determine its long-term plausibility.

### Increased outdoor transmission rates and widespread insecticide resistance threaten conventional malaria vector control approaches.

The selected findings here demonstrated that active indoor and outdoor residual malaria transmission were comparable across different ecological settings.^22^ The variable endophilic behavior of *A. gambiae* s.l. relative to human outdoor nocturnal behavior and more extended outdoor dwelling periods could explain the high observed outdoor biting rates and EIRs. Other environmental factors, particularly elevated ambient temperature,^20^ showed insufficient protection against outdoor mosquitoes. Increased LLIN use may further explain these phenomena. Notable challenges to vector control were the multiple resistance mechanisms to pyrethroids observed in *A. gambiae* s.l., displaying a widespread and high phenotypic resistance to deltamethrin and permethrin.^29^^,^^30^ The high outdoor transmission patterns and widespread insecticide resistance highlight the need for additional strategies to combat outdoor malaria transmission. These strategies should complement traditional indoor preventive approaches, such as the next generation of LLINs^35^ and attractive targeted sugar baits, which focus on outdoor mosquitoes.^36^^,^^37^ The WA-ICEMR studies also described seasonal spatiotemporal clusters in the distribution of *P. falciparum* infection and diseases that may provide opportunities for new control strategies such as reactive, community-based, self-administered treatments against residual malaria transmission or targeted vector control strategies such as larval control of vector breeding and during the dry season.^26^^,^^38^ These studies also showed that spatial analyses and cluster detection approaches have considerable utility for guiding targeted intervention strategies.

## CONCLUSION

The WA-ICEMR studies revealed that the expansion and enhancement of ongoing intervention strategies such as LLINs and SMC have significantly reduced the malaria burden in Mali. Despite this progress, the persistence of residual human reservoirs of parasite carriers and circulating malaria vectors accentuate the critical need to jointly maintain current malaria control strategies and develop innovative approaches to further disrupt malaria transmission. Findings from implementation studies may drive policy for SMC treatment options, such as potentially expanding its age targets and developing alternative preventive drug regimens. However, there is a critical need for strengthening molecular surveillance for insecticide and drug resistance to monitor the effectiveness of control interventions, including universal LLIN coverage and SMC strategies and mitigate the impact of potential emerging resistance. The partnership between the WA-ICEMR and NMCP provides a highly effective model for maximizing the impact of sponsored research projects. This partnership was particularly useful in establishing goal-based platforms for research while leveraging resources and enhancing governmental outreach activities.

## References

[1] Trape JF 2014. The rise and fall of malaria in a West African rural community, Dielmo, Senegal, from 1990 to 2012: a 22 year longitudinal study. Lancet Infect Dis 14: 476–488. 24813159 10.1016/S1473-3099(14)70712-1

[2] Bhatt S 2015. The effect of malaria control on *Plasmodium falciparum* in Africa between 2000 and 2015. Nature 526: 207–211. 26375008 10.1038/nature15535 PMC4820050

[3] WHO , 2020. World Malaria Report 2020. Geneva, Switzerland: World Health Organization.

[4] Mwesigwa J Okebe J Affara M Di Tanna GL Nwakanma D Janha O Opondo K Grietens KP Achan J D’Alessandro U , 2015. On-going malaria transmission in The Gambia despite high coverage of control interventions: a nationwide cross-sectional survey. Malar J 14: 314. 26268225 10.1186/s12936-015-0829-6 PMC4535679

[5] Coulibaly D 2014. Stable malaria incidence despite scaling up control strategies in a malaria vaccine-testing site in Mali. Malar J 13: 374. 25238721 10.1186/1475-2875-13-374 PMC4180968

[6] Cellule de Planification et de Statistique—CPS/SSDSPF/Mali, Institut National de la Statistique—INSTAT/Mali, Centre d’Études et d’Information Statistiques—INFO-STAT/Mali, ICF International , 2014. *Mali Enquête Démographique et de Santé (EDSM V) 2012–2013*. Rockville, MD: CPS, INSTAT, INFO-STAT and ICF International.

[7] Jagannathan P Muhindo MK Kakuru A Arinaitwe E Greenhouse B Tappero J Rosenthal PJ Kaharuza F Kamya MR Dorsey G , 2012. Increasing incidence of malaria in children despite insecticide-treated bed nets and prompt anti-malarial therapy in Tororo, Uganda. Malar J 11: 435. 23273022 10.1186/1475-2875-11-435 PMC3551700

[8] Rao MR , 2015. Foreword: international centers of excellence for malaria research. Am J Trop Med Hyg 93: 1–4. 10.4269/ajtmh.15-0407 PMC4574268 26574613

[9] Ceesay SJ 2012. Sahel, savana, riverine and urban malaria in West Africa: similar control policies with different outcomes. Acta Trop 121: 166–174. 22119584 10.1016/j.actatropica.2011.11.005 PMC3294051

[10] Doumbia SO 2012. Improving malaria control in West Africa: interruption of transmission as a paradigm shift. Acta Trop 121: 175–183. 22142790 10.1016/j.actatropica.2011.11.009 PMC3294075

[11] Programme National de Lutte contre le Paludisme (PNLP), Institut National de la Statistique (INSTAT) I-S, Institut National de la Recherche en Santé Publique (INRSP), ICF International , 2016. *Enquête sur les Indicateurs du Paludisme au Mali (EIPM) 2015.* Rockville, MD: INSTAT, INFO-STAT et ICF International.

[12] Severe Malaria Observatory , n.d. *Mali: Malaria Facts*. Available at: https://www.severemalaria.org/countries/mali#:~:text=A%20new%20malaria%20control%20program,to%20eliminate%20malaria%20by%202030. Accessed March 16, 2022.

[13] Mitchell S 2018. The Millennium Villages Project: a retrospective, observational, endline evaluation. Lancet Glob Health 6: e500–e513. 29653625 10.1016/S2214-109X(18)30065-2

[14] United Nations Development Programme , 2009. *The Millennium Villages Project 2009 Annual Report*. Available at: https://irp-cdn.multiscreensite.com/6fae6349/files/uploaded/MVP%202009%20Annual%20Report%20-%20EIMPUNDP%20-%20General%20Public%20Version%20-%20FINAL.pdf. Accessed March 15, 2022.

[15] Tholander S , 2011. *Trees for Livelihoods: Insights into Practice and Adoption of Agroforestry in Tiby, Mali*. Available at: https://citeseerx.ist.psu.edu/viewdoc/download?doi=10.1.1.840.474&rep=rep1&type=pdf. Accessed March 15, 2022.

[16] Sachs JD , 2018. Lessons from the Millennium Villages Project: a personal perspective. Lancet Glob Health 6: e472–e474. 29653613 10.1016/S2214-109X(18)30199-2

[17] UN Millennium Project , 2005. *Investing in Development: A Practical Plan to Achieve the Millennium Development Goals*. Available at: http://siteresources.worldbank.org/INTTSR/Resources/MainReportComplete-lowres%5B1%5D.pdf. Accessed March 15, 2022.

[18] Shaffer JG 2018. Development of a data collection and management system in West Africa: challenges and sustainability. Infect Dis Poverty 7: 125. 30541626 10.1186/s40249-018-0494-4 PMC6292095

[19] Shaffer JG 2020. Clustering of asymptomatic *Plasmodium falciparum* infection and the effectiveness of targeted malaria control measures. Malar J 19: 33. 31964378 10.1186/s12936-019-3063-9 PMC6975028

[20] Ateba FF 2020. Spatio-temporal dynamic of malaria incidence: a comparison of two ecological zones in Mali. Int J Environ Res Public Health 17: 4698. 10.3390/ijerph17134698 PMC7370019 32629876

[21] Kané F Keïta M Traoré B Diawara SI Bane S Diarra S Sogoba N Doumbia S , 2020. Performance of IRS on malaria prevalence and incidence using pirimiphos-methyl in the context of pyrethroid resistance in Koulikoro region, Mali. Malar J 19: 286. 32787938 10.1186/s12936-020-03357-8 PMC7425591

[22] Keïta M 2021. Indoor and outdoor malaria transmission in two ecological settings in rural Mali: implications for vector control. Malar J 20: 127. 33663515 10.1186/s12936-021-03650-0 PMC7931590

[23] Diakite SAS 2019. A comprehensive analysis of drug resistance molecular markers and *Plasmodium falciparum* genetic diversity in two malaria endemic sites in Mali. Malar J 18: 361. 31718631 10.1186/s12936-019-2986-5 PMC6849310

[24] Traore K 2020. Ex-vivo sensitivity of *Plasmodium falciparum* to common anti-malarial drugs: the case of Kenieroba, a malaria endemic village in Mali. Drugs R D 20: 249–255. 32557086 10.1007/s40268-020-00313-4 PMC7419409

[25] Touré M 2022. Trends in malaria epidemiological factors following the implementation of current control strategies in Dangassa, Mali. Malar J 21: 65. 35197053 10.1186/s12936-022-04058-0 PMC8867639

[26] Ateba FF 2020. Predicting malaria transmission dynamics in Dangassa, Mali: a novel approach using functional generalized additive models. Int J Environ Res Public Health 17: 6339. 10.3390/ijerph17176339 PMC7504016 32878174

[27] Konaté D 2021. Effectiveness and community acceptance of extending seasonal malaria chemoprevention to children 5 to 14 years of age in Dangassa, Mali. Am J Trop Med Hyg 106: 648–654. 34781256 10.4269/ajtmh.21-0046 PMC8832934

[28] Müller GC Beier JC Traore SF Toure MB Traore MM Bah S Doumbia S Schlein Y , 2010. Field experiments of *Anopheles gambiae* attraction to local fruits/seedpods and flowering plants in Mali to optimize strategies for malaria vector control in Africa using attractive toxic sugar bait methods. Malar J 9: 262. 20854666 10.1186/1475-2875-9-262 PMC2949744

[29] Keïta M Sogoba N Traoré B Kané F Coulibaly B Traoré SF Doumbia S , 2021. Performance of pirimiphos-methyl based indoor residual spraying on entomological parameters of malaria transmission in the pyrethroid resistance region of Koulikoro, Mali. Acta Trop 216: 105820. 33400915 10.1016/j.actatropica.2020.105820 PMC8008285

[30] Keita M Sogoba N Kane F Traore B Zeukeng F Coulibaly B Sodio AB Traore SF Djouaka R Doumbia S , 2021. Multiple resistance mechanisms to pyrethroids insecticides in *Anopheles gambiae* sensu lato population from Mali, West Africa. J Infect Dis 223: S81–S90. 33906223 10.1093/infdis/jiaa190 PMC8079131

[31] Keita M Kane F Thiero O Traore B Zeukeng F Sodio AB Traore SF Djouaka R Doumbia S Sogoba N , 2020. Acetylcholinesterase (ace-1(R)) target site mutation G119S and resistance to carbamates in *Anopheles gambiae* (sensu lato) populations from Mali. Parasit Vectors 13: 283. 32503614 10.1186/s13071-020-04150-x PMC7275337

[32] Sovi A 2020. *Anopheles gambiae* (s.l.) exhibit high intensity pyrethroid resistance throughout southern and central Mali (2016–2018): PBO or next generation LLINs may provide greater control. Parasit Vectors 13: 239. 32384907 10.1186/s13071-020-04100-7 PMC7206711

[33] Gutman J Kovacs S Dorsey G Stergachis A Ter Kuile FO , 2017. Safety, tolerability, and efficacy of repeated doses of dihydroartemisinin-piperaquine for prevention and treatment of malaria: a systematic review and meta-analysis. Lancet Infect Dis 17: 184–193. 27865890 10.1016/S1473-3099(16)30378-4 PMC5266794

[34] Uwimana A 2020. Emergence and clonal expansion of in vitro artemisinin-resistant *Plasmodium falciparum* kelch13 R561H mutant parasites in Rwanda. Nat Med 26: 1602–1608. 32747827 10.1038/s41591-020-1005-2 PMC7541349

[35] WHO , 2014. *World Malaria Report 2014*. Geneva, Switzerland: World Health Organization.

[36] Diarra RA 2021. Testing configurations of attractive toxic sugar bait (ATSB) stations in Mali, West Africa, for improving the control of malaria parasite transmission by vector mosquitoes and minimizing their effect on non-target insects. Malar J 20: 184. 33853632 10.1186/s12936-021-03704-3 PMC8048058

[37] Fraser KJ 2021. Estimating the potential impact of Attractive Targeted Sugar Baits (ATSBs) as a new vector control tool for *Plasmodium falciparum* malaria. Malar J 20: 151. 33731111 10.1186/s12936-021-03684-4 PMC7968277

[38] Okebe J 2021. Reactive, self-administered malaria treatment against asymptomatic malaria infection: results of a cluster randomized controlled trial in The Gambia. Malar J 20: 253. 34098984 10.1186/s12936-021-03761-8 PMC8186162

